# Decoding Individual Episodic Memory Traces in the Human Hippocampus

**DOI:** 10.1016/j.cub.2010.01.053

**Published:** 2010-03-23

**Authors:** Martin J. Chadwick, Demis Hassabis, Nikolaus Weiskopf, Eleanor A. Maguire

**Affiliations:** 1 Wellcome Trust Centre for Neuroimaging, Institute of Neurology, University College London, 12 Queen Square, London WC1N 3BG, UK

**Keywords:** SYSNEURO

## Abstract

In recent years, multivariate pattern analyses have been performed on functional magnetic resonance imaging (fMRI) data, permitting prediction of mental states from local patterns of blood oxygen-level-dependent (BOLD) signal across voxels [1, 2]. We previously demonstrated that it is possible to predict the position of individuals in a virtual-reality environment from the pattern of activity across voxels in the hippocampus [3]. Although this shows that spatial memories can be decoded, substantially more challenging, and arguably only possible to investigate in humans [4], is whether it is feasible to predict which complex everyday experience, or episodic memory, a person is recalling. Here we document for the first time that traces of individual rich episodic memories are detectable and distinguishable solely from the pattern of fMRI BOLD signals across voxels in the human hippocampus. In so doing, we uncovered a possible functional topography in the hippocampus, with preferential episodic processing by some hippocampal regions over others. Moreover, our results imply that the neuronal traces of episodic memories are stable (and thus predictable) even over many re-activations. Finally, our data provide further evidence for functional differentiation within the medial temporal lobe, in that we show the hippocampus contains significantly more episodic information than adjacent structures.

## Highlights

► It is possible to decode specific episodic memory traces from fMRI activity ► Individual memories can be decoded from fMRI activity in the human hippocampus (HC) ► The HC contains significantly more episodic information than adjacent brain areas ► Episodic information is localized to bilateral anterior and right posterior HC

## Results and Discussion

The search for the elusive engram, or memory trace, in the brain has been an ongoing endeavor in neuroscience for nearly a century [5–7]. Although the biological existence of such engrams coding for memories is widely accepted, the precise mechanisms, locations, and even nature of the engram itself, in light of processes such as reconsolidation [7, 8], is the subject of much debate. The components of a complex multimodal memory, such as a rich episodic memory, are likely to be widely distributed throughout the cortex [9]. These components on their own are not sufficient, however. Something must bind the disparate elements of a recent episodic memory together to allow the relevant neural representations to coactivate, thus facilitating recollection [10]. Marr [11] proposed that the hippocampus provides this function by storing a memory “index,” a distilled representation containing the essence of the memory, which is synaptically linked to the full representation stored in the neocortex. The hippocampus is ideally suited for multimodal binding, given its purported location at the top of the sensory cortical hierarchy and its widely acknowledged role in supporting episodic memory [12, 13].

Precisely how the hippocampus codes for episodic memories, however, is still unknown. This is because tracking an individual episodic memory in terms of the activity of the many thousands of hippocampal neurons that support it remains a substantial challenge [3, 14], complicated further by the possibility that episodic memories might be uniquely human [4]. Multivariate pattern analysis (MVPA) techniques applied to human functional magnetic resonance imaging (fMRI) data [1, 2] may offer a means to bridge the gap between recordings from single neurons and examining episodic memory across large populations of neurons in humans. MVPA assesses local patterns of information across voxels, permitting the differentiation of distinct perceptual and mental states in a manner not possible via conventional univariate fMRI analyses [1, 2]. In a recent study, MVPA was used to decode spatial information and predict the location of participants in a virtual-reality environment from the pattern of fMRI signals across voxels in the human hippocampus [3]. Here, using high-spatial-resolution fMRI, we investigated whether it would be possible to predict which specific recent episodic memory a participant was recalling solely on the basis of the blood oxygen-level-dependent (BOLD) activity patterns across voxels in the hippocampus, thus potentially distinguishing specific memory traces.

**Figure 1:**
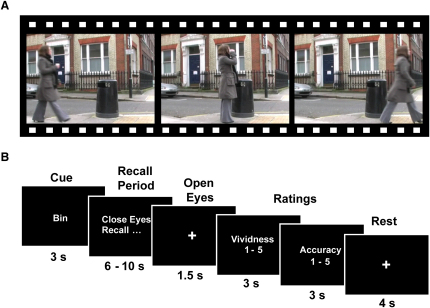
Experimental Protocol (A) Still photographs taken from one of the film clips viewed during prescan training. The clip depicts a woman taking a drink from a disposable coffee cup and then putting it in a rubbish bin (trash can). (B) Timeline of a single trial during fMRI scanning. For details, see Supplemental Experimental Procedures.

In a prescan training session, ten participants repeatedly viewed short film clips of three distinct everyday events (Figure 1A). During fMRI scanning, a participant was required to vividly recall in as much detail as possible each of the three episodes a number of times (Figure 1B). We applied a multivariate decoding technique, based on a linear support vector machine (SVM) [15] with multivariate feature selection [16], to the fMRI signals within the hippocampus (see Experimental Procedures and the Supplemental Experimental Procedures available online). This analysis revealed episodic memory decoding in the hippocampus for every participant, showing that it is possible to predict which specific episodic memory was being recalled solely from the pattern of fMRI BOLD signals across voxels in the hippocampus.

**Figure 2:**
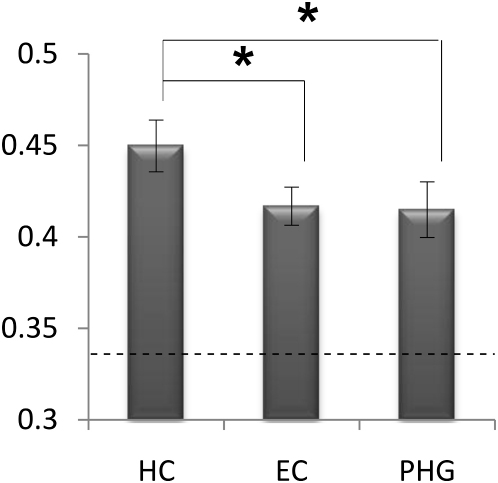
Mean Decoding Accuracy Results with Standard Errors for the Hippocampus, Entorhinal Cortex, and Parahippocampal Gyrus Proportion accuracy values are shown on the vertical axis; the dashed line at 0.33 represents chance-level performance. All three areas were significantly above chance-level performance, with hippocampus (HC) accuracy significantly greater than both entorhinal cortex (EC) and parahippocampal gyrus (PHG) (one-way analysis of variance, p = 0.027; post hoc t tests: HC > EC, p = 0.035; HC > PHG, p = 0.048; no significant difference between EC and PHG, p = 0.86). See Supplemental Experimental Procedures and Figure S1 for more details. Error bars represent the standard error of the mean.

Given that the entorhinal cortex (EC) and posterior parahippocampal gyrus (PHG) are both major input pathways to the hippocampus [17], we then investigated whether these regions might also contain episodic information. The same analysis techniques were applied to EC and PHG, and results showed that both of these areas contained episodic information (Figure 2). However, this information was significantly reduced compared with the hippocampus (HC). Thus, not only is it possible to decode individual episodic memories from all three medial temporal lobe regions, but the relative degree of decoding reflects the anatomical and functional hierarchy of these areas [18].

**Figure 3:**
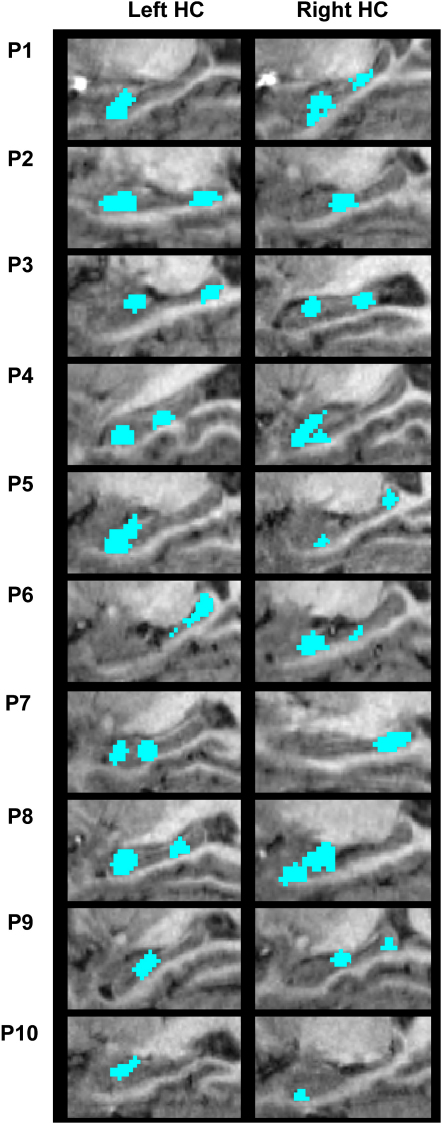
Individual Participant Data Hippocampal information maps in the left and right hippocampi are shown for the ten participants (P1–P10) on zoomed-in sagittal sections of the medial temporal lobes taken from each participant's structural MRI scan. Each map represents the set of voxels carrying the most episodic information within the hippocampus. See Supplemental Experimental Procedures and Figure S2 for more details.

**Figure 4:**
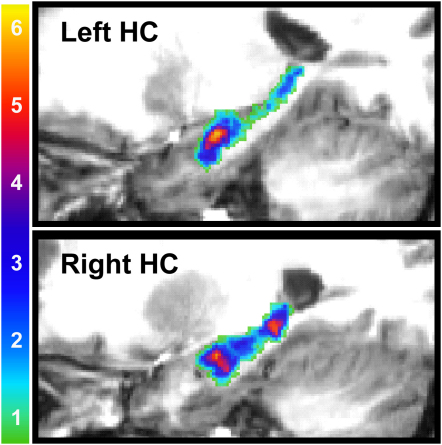
Consistency of Results across Participants Frequency heat maps for the left and right hippocampi shown on zoomed-in sagittal sections from one of the participant's structural MRI scans chosen at random. Frequency scale is shown at the left. To determine statistical significance, we compared the frequency value at each voxel against the binomial distribution, and the peak regions in yellow and red all survived an uncorrected p < 0.001 level of significance.

A priori, it is not clear whether particular regions within the hippocampus should show a preference for coding individual episodic memories. A useful property of the feature selection method used in this analysis is that it produced a subset of voxels within a region of interest that carried the most episodic information (see Experimental Procedures and Supplemental Experimental Procedures). We refer to this as the “information map” for that region, and the hippocampal information maps for all ten participants are displayed in Figure 3. An inspection of these maps suggests that there may be consistencies across participants in the location of episodic information. To examine this further, we transformed the hippocampal information maps for all ten participants into standard stereotactic space and added them together to form a frequency heat map (Figure 4). This heat map clearly shows three peak regions of overlap, in bilateral anterior and right posterior hippocampus. To quantify these results, we compared the frequency count at each voxel (out of a maximum frequency of 10) against the binomial distribution and derived a p value. All three peak regions in red and yellow are significant at a threshold of p = 0.001. This result demonstrates that episodic information is not randomly distributed across the hippocampus but is instead concentrated within specific regions.

In summary, we have documented for the first time that traces of individual rich episodic memories are detectable and distinguishable in the human hippocampus. Moreover, our results show remarkable consistency across participants and suggest a functional topography in the hippocampus, with preferential episodic processing by some hippocampal regions over others. We speculate that the involvement of the right posterior hippocampus may relate to the coding of spatial locations in the memories [3, 19], while the robust loci in bilateral anterior hippocampal regions are consistent with previous studies of autobiographical memory [20] and represent a clear target for future investigations. Another striking feature of our findings is the stability of the memory traces. The MVPA classifier could only successfully decode hippocampal activity if the differences between the memories were systematic and consistent across the majority of the training examples. Thus, our results imply that the neuronal traces of the memories were stable even over many re-activations. Finally, our data provide further evidence for functional differentiation within the medial temporal lobe, with the hippocampus containing significantly more episodic information than adjacent structures.

Now that we have shown that it is possible to directly access information about individual episodic memories in the human hippocampus in vivo and noninvasively, this offers new opportunities to examine important properties of episodic memory, to explore possible functional topographies, and to examine neural computations within hippocampal subfields [21].

## Experimental Procedures

### Participants

Ten healthy right-handed participants (six female, four male) took part in the experiment (mean age 21.1 years, standard deviation 1.8 years, range 18–24 years). All had normal or corrected-to-normal vision and gave informed written consent to participation in accordance with the local research ethics committee.

### Task

During a prescan training period, participants viewed three film clips of everyday events. Each clip was 7 s long and featured a woman (a different woman in each clip) carrying out a short series of actions (see Supplemental Experimental Procedures). Each participant viewed each clip 15 times and practiced vividly recalling them. During scanning, there were two experimental conditions. The first involved a cued recall task where on each trial, the participant was presented with a cue indicating which of the three film events they were required to recall (see Figure 1). Following this, an instruction appeared on the screen indicating that the participant should close their eyes and vividly recall the cued memory. The cued recall condition contained a total of 21 trials, with seven trials of each memory, presented in a pseudorandom order while ensuring that the same memory was not repeated two or more times in a row. The second condition was a free recall task where the participant was allowed to decide which of the three episodes they would recall on each trial (for the statistical dependencies that result from this free choice behavior, see Table S1). Here, the cue period was replaced with a decision period, during which the participant decided which of the three memories they would subsequently recall, and following recall, the participants were required to indicate via an MRI-compatible keypad which of the three memories they had just recollected. The free recall condition included a total of 30 trials, and participants were instructed to sample from the three memories. For each cued and free recall trial, participants then performed a series of ratings (see Figure 1B, Supplemental Experimental Procedures, and Table S2). Both experimental conditions were scanned in a single functional run, starting with the cued recall condition, with a 30 s rest period before the free recall condition. Multivariate pattern analyses using the cued or free recall trials separately yielded significant decoding results in all three anatomical regions, and there were no significant differences between the two conditions (see Supplemental Experimental Procedures), demonstrating that decoding does not depend on the specific retrieval mode. For all subsequent analyses, the data were collapsed across both conditions in order to investigate patterns of information that held across different retrieval modes. These are the results reported above. After the scanning session, participants completed a debriefing questionnaire that was designed to assess various factors such as the emotional response to each memory and similarity to real memories (see Supplemental Experimental Procedures for the full questionnaire and Table S3 for mean scores and analyses). There were no significant differences between the three memories for these ratings, making it unlikely that these extraneous factors could have driven the decoding performance.

### Image Acquisition

A 3T Magnetom Allegra head-only MRI scanner (Siemens Medical Solutions) operated with the standard transmit-receive head coil was used to acquire functional data with a T2^∗^-weighted single-shot echo-planar imaging sequence (in-plane resolution = 1.5 × 1.5 mm^2^, matrix = 128 × 128, field of view = 192 × 192 mm^2^, 35 slices acquired in interleaved order, slice thickness = 1.5 mm with no gap between slices, echo time [TE] = 30 ms, asymmetric echo shifted forward by 26 phase-encoding lines, echo spacing = 560 μs, repetition time [TR] = 3.5 s, flip angle α = 90°). All data were acquired at 0° angle in the anterior-posterior axis in one single uninterrupted functional scanning session. An isotropic voxel size of 1.5 × 1.5 × 1.5 mm^3^ was chosen for an optimal tradeoff between BOLD sensitivity and spatial resolution. Furthermore, the isotropic voxel dimension reduced resampling artifacts when applying motion correction. For distortion correction [22], field maps were acquired with a standard manufacturer's double-echo gradient echo field map sequence (TE = 10.0 and 12.46 ms, TR = 1020 ms, matrix size = 64 × 64), with 64 slices covering the whole head (voxel size = 3 × 3 × 3 mm^3^). A T1-weighted high-resolution 3D modified driven equilibrium Fourier transform whole-brain structural MRI scan was acquired for each participant after the main scanning session with 1 mm isotropic resolution [23].

### Image Preprocessing for Multivariate Analysis

T1-weighted structural images were anatomically segmented with the FreeSurfer automated cortical and subcortical parcellation tools [24, 25]. This generated a set of hippocampus, entorhinal cortex, and parahippocampal gyrus masks for each participant, which were then manually corrected where necessary to ensure that they were in line with the anatomical guidelines set out by Insausti et al. [26]. A linear detrend was applied to the data with a first-order polynomial function. The procedure was performed by fitting the linear drift to the whole run and subtracting it from the voxel intensities. The onset times were then shifted to account for the delay in hemodynamic response [2].

### Multivariate Classification

In order to assess the degree of episodic information contained within medial temporal lobe structures, we used a two-step procedure incorporating first feature selection and then final multivariate classification [16]. The purpose of feature selection is to reduce the set of features (in this case, voxels) in a data set to those most likely to carry relevant information. The particular feature selection strategy employed was a multivariate searchlight strategy, which assesses the local pattern of information surrounding each voxel in turn [27] (see Supplemental Experimental Procedures and Figure S2 for more details). The overall classification procedure involved splitting the imaging data into two segments: a “training” set used to train a linear support vector machine (with fixed regularization hyperparameter C = 1) in order to identify response patterns related to the memories being discriminated, and a “test” set used to independently test the classification performance (see Supplemental Experimental Procedures and Figure S1 for more details). The SVM classifier was trained to discriminate between the three memories with the “training” image data set and tested on the independent “test” data set. The classification was performed with the LIBSVM (http://www.csie.ntu.edu.tw/∼cjlin/libsvm/) implementation. We used a standard k-fold cross-validation testing regime [15] wherein k equaled the number of experimental trials. Note that standard SVMs are binary classifiers that operate on two-class discrimination problems. The SVM can, however, be arbitrarily extended to work in cases where there are more than two classes, such as the three memories in this study (see Supplemental Experimental Procedures).

## Supplemental Information

Document S1. Two Figures, Three Tables, and Supplemental Experimental Procedures
