# Supplementary material for: Decoding Individual Episodic Memory Traces in the Human Hippocampus
Source: Curr Biol. 2010 Mar 23;20(6):544–7. doi: 10.1016/j.cub.2010.01.053 (PMC2849012; doi:10.1016/j.cub.2010.01.053)
Supplement: Document S1. Two Figures, Three Tables, and Supplemental Experimental Procedures [file mmc1.pdf]

Current Biology, Volume *20*

Supplemental Information

Decoding Individual Episodic Memory Traces in the Human Hippocampus

Martin J. Chadwick, Demis Hassabis, Nikolaus Weiskopf, and Eleanor A. Maguire

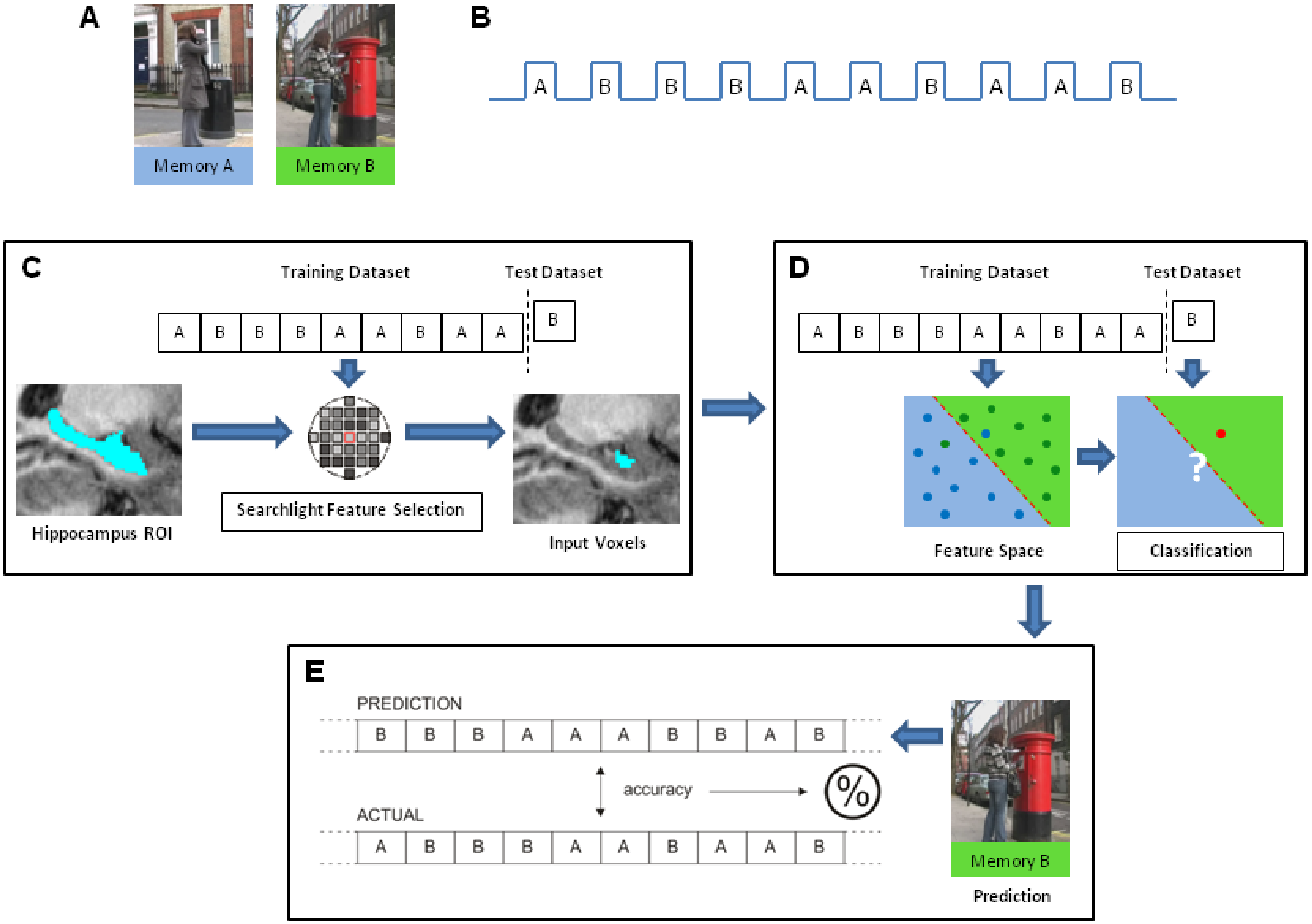
A
Memory A
Memory B
B
A B B B A A B A A B
C
Training Dataset
Test Dataset
A B B B A A B A A
B
Hippocampus ROI
Searchlight Feature Selection
Input Voxels
D
Training Dataset
Test Dataset
A B B B A A B A A
B
Feature Space
?
Classification
E
PREDICTION
B B B A A A B B A B
accuracy
%
ACTUAL
A B B B A A B A A B
Memory B
Prediction

Figure S1.

Figure S1. Illustration of the Multivariate Classification Procedure

(A) For simplicity we demonstrate the procedure when classifying two distinct episodic memories, while in reality we classified three memories. In this case Memory A involved a woman sipping from a disposable coffee cup and putting into a rubbish bin (trashcan), and Memory B involved a woman posting a letter into a postbox (mailbox).

(B) Only volumes acquired during the memory recall period of each trial were entered into the analysis.

(C) The full data set was split into a “training” set and a “test” set, where the test set was the data from a single experimental trial. Using the training set, searchlight feature selection was applied to the voxels within the region of interest (ROI), in this example the hippocampus (see Supplemental Experimental Procedures and Figure S2 for details). This resulted in a reduced set of voxels which carried the most information.

(D) Using the reduced voxel set, a classifier was trained to differentiate memories A and B using the training data set, and then tested using the fully independent test set.

(E) In this case the test trial was classified as Memory B, which was a correct prediction. A standard k-fold cross-validation testing regime was implemented, ensuring that all trials were used once as the test data set. This cross-validation therefore yielded a predicted label for every data trial in the analysis, which was then compared to the real labels to produce and overall prediction accuracy value.

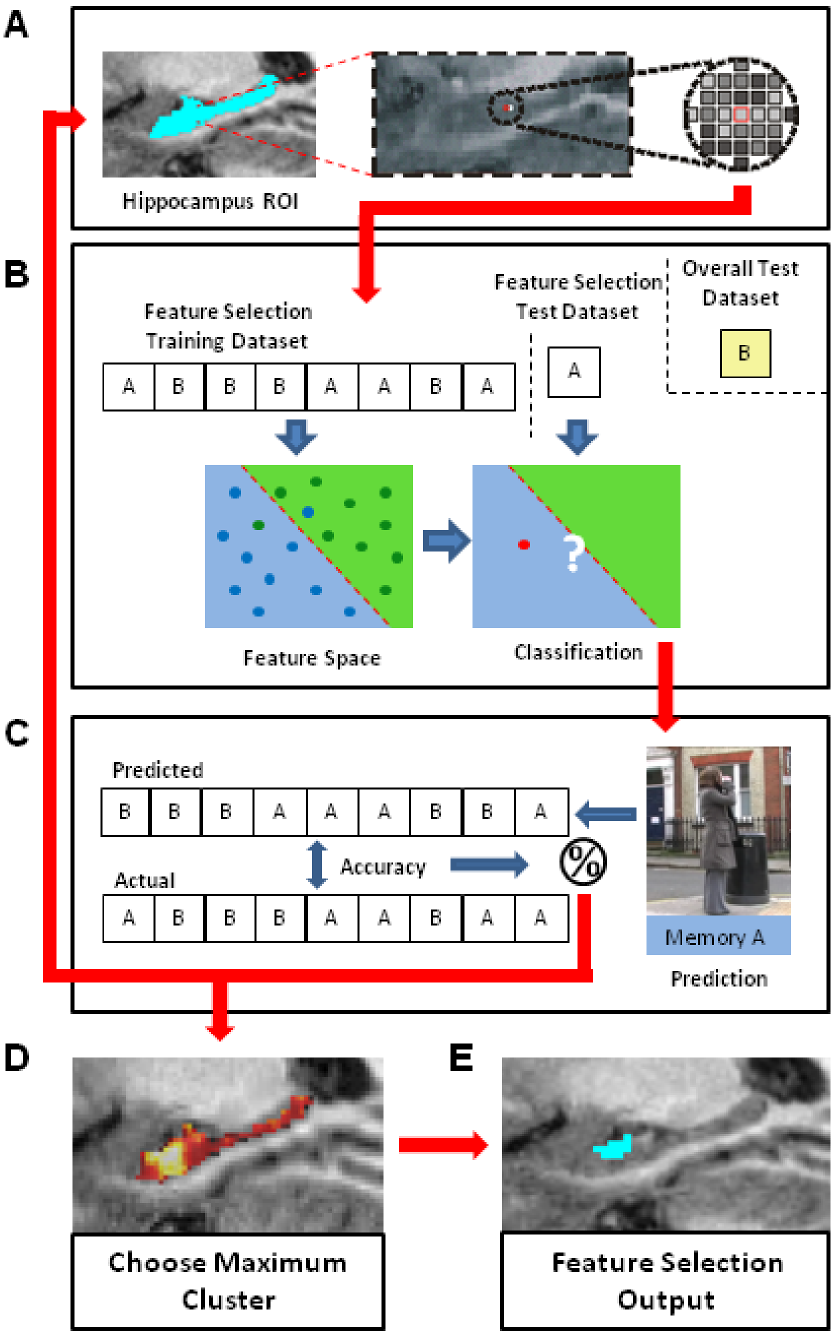

Figure S2. Illustration of the Searchlight Feature Selection Procedure

(A) The searchlight analysis stepped through every single voxel in the search space, which was defined by an anatomical ROI, in this example the hippocampus. For each voxel (example outlined in red), a spherical cluster (radius 3 voxels) of 99 voxels was extracted from around this central voxel.

(B) Once the overall test data set had been removed (see Supplemental Experimental Procedures and Figure S1), the remaining feature selection data was separated into a training set and a test set (which was the data from a single experimental trial). Using the voxel cluster from the searchlight, a classifier was trained to differentiate memories A and B using the training data, and then tested using the independent feature selection test data.

(C) In this case the test trial was classified as Memory A, which was a correct prediction. A standard k-fold cross-validation testing regime was implemented, ensuring that all data trials were used once as the test data set. This cross-validation therefore yielded a predicted label for every trial in the analysis, which was then compared to the real labels to produce an overall prediction accuracy value. The whole procedure was then repeated for every single voxel within the search space.

(D) This created an "accuracy map" of the whole ROI, with an accuracy value at each voxel representing the amount of information contained within the searchlight sphere surrounding that voxel. Here the accuracy values for each voxel are displayed in a heat map.

(E) For the feature selection output, the searchlight cluster with the highest accuracy value, and therefore greatest amount of information, was chosen and it is this voxel set that was fed into the overall classification analysis.

**Table S1.**						
	**AB**	**AC**	**BA**	**BC**	**CA**	**CB**
**P1**	0.45	0.55	0.56	0.44	0.67	0.33
**P2**	0.29	0.71	0.44	0.56	0.30	0.70
**P3**	0.63	0.38	0.55	0.45	0.25	0.75
**P4**	0.88	0.13	0.22	0.78	0.75	0.25
**P5**	0.44	0.56	0.50	0.50	0.56	0.44
**P6**	0.44	0.56	0.60	0.40	0.33	0.67
**P7**	0.44	0.56	0.50	0.50	0.40	0.60
**P8**	1.00	0.00	0.10	0.90	0.88	0.13
**P9**	0.73	0.27	0.64	0.36	0.43	0.57
**P10**	0.50	0.50	0.67	0.33	0.86	0.14
**Mean**	**0.58**	**0.42**	**0.48**	**0.52**	**0.54**	**0.46**
**SD**	**0.22**	**0.22**	**0.18**	**0.18**	**0.23**	**0.23**

Pairwise statistical dependencies displayed by each participant (P1 – P10) during the Free Recall condition, along with group mean and standard deviation. Column 2 displays the probability that the recall of memory A was followed by the recall of memory B, column 3 displays the probability of memory C following memory A, and so on. Note that participants were explicitly instructed *not* to recall the same memory twice in a row; therefore the probably of each memory being followed by itself is zero. Some participants display strong dependencies, but the group as a whole was well balanced, and none of the group dependencies was significantly different from chance (50%).

**Table S2. Summary of Participants' Behavioral Performance during Scanning**			
	**Postbox**	**Bicycle**	**Bin**
**Number of trials**	14.5 (1.27)	14.6 (2.88)	13.7 (2.63)
**Recall Length (s)**	7.74 (0.2)	7.98 (0.51)	7.94 (0.43)
**Vividness (1-5)**	4.01 (0.56)	4.01 (0.55)	4.02 (0.61)
**Accuracy (1-5)**	4.01 (0.53)	4.01 (0.53)	3.99 (0.63)

Means for number of trials, length of recall period, vividness, and accuracy ratings are displayed for each of the three memories collapsed across both the cued and free recall tasks. Standard deviations are displayed in parentheses. These summary statistics were derived *after* exclusion of low rating trials and trials that were too long or too short (see Supplemental Experimental Procedures). For each of these variables, a repeated measures ANOVA was applied to determine if there were consistent differences between the three memories. None of these analyses found any significant results.

**Table S3. Summary of Debriefing Questionnaire Results**			
	**Postbox**	**Bicycle**	**Bin**
**Difficulty**	2.2 (0.79)	2.3 (0.95)	1.9 (1.1)
**Emotionality**	1 (0)	1 (0)	1.2 (0.42)
**Similarity to real memory**	1.7 (0.82)	1.7 (0.82)	1.6 (0.84)
**Thinking about self**	1.5 (0.71)	1.4 (0.7)	1.7 (0.82)
**Perspective-taking**	1.9 (0.88)	1.2 (0.42)	1.8 (1.03)
**Background story**	2 (1.49)	1.3 (0.48)	1.6 (0.97)

Mean ratings are provided for each of the three memories for each questionnaire item, with standard deviations in parentheses. Participants were asked to provide ratings on a scale of 1 – 5 (low – high). For each of these variables, a repeated measures ANOVA was applied to determine if there were consistent differences between the three memories. None of these analyses found any significant differences.

Supplemental Experimental Procedures

Prescan Training

During a prescan training period, participants viewed three film clips of everyday events. Each clip was 7s long and featured a woman (a different woman in each clip) carrying out a short series of actions. The films were shot outdoors in three different urban settings. These stimuli ensured that memories would be episodic-like in nature, and that all participants recalled the same set of memories. One clip featured a woman taking a letter out of her handbag, posting it in a post box (mailbox), and then walking off. Another clip featured a woman taking a drink from a disposable coffee cup, putting the cup in a rubbish bin (trashcan), and then walking off (see Figure 1A). The final clip featured a woman picking up a bicycle that was leaning against some railings, adjusting her helmet and walking off with the bicycle. A participant saw each clip 15 times, and practised vividly recalling them. A further consideration was the length of time it took to recall the memory of a clip. As each memory would be recalled multiple times in the scanning session (on average 17 times), it was important that the temporal duration of the recall period was similar on each occasion. This temporal dimension was therefore emphasised during training, and feedback was provided on the timing accuracy on each practice trial. This extensive training ensured that the duration of recall was consistent for each memory and across the three memories.

Task

There were two experimental conditions during scanning. The first involved a cued recall task where on each trial the participant was presented with a cue indicating which of the three film events they were required to recall (see Figure 1). Following this, an instruction appeared on the screen indicating that the participant should close their eyes and vividly recall the cued memory. Participants were instructed not to begin the recall process until this instruction appeared, and were trained on this procedure in the prescan session. At this point we included a check that the participants were concentrating, and to make sure that the recall approximated the original 7s length of a clip. The participant had to press a button (using a scanner-compatible button-box) when they had finished recalling the clip. If the button was pushed too soon (<6s) or they failed to push it within 10s then the participant would hear a tone, and a message would appear for 1.5s indicating that their recall had been too fast or too slow. Any such trials were excluded from the subsequent analysis. If the participant pressed the button between 6-10s, a fixation cross appeared onscreen for 1.5s. Participants were trained to open their eyes as soon as they had pressed the button or if they heard a tone. Following this, the participant was required to provide ratings about the preceding recall trial using the five-key button-box. Firstly, they rated how vivid the preceding recall trial was (scale: 1 – 5, where 1 was not vivid at all, and 5 was extremely vivid). Secondly, they rated how accurately the recalled memory reflected the actual film clip (scale: 1 – 5, where 1 was not accurate at all, and 5 was extremely accurate). Any trials where a participant recorded a rating of less than 3 were excluded from the subsequent analysis. Following the ratings, participants rested for 4s before starting the next trial. The cued recall condition contained a total of 21 trials, with seven trials of each memory, presented in a pseudo-random order, whilst ensuring that the same memory was not repeated twice or more in a row.

The second condition was a free recall task, where the participant was allowed to decide which of the three episodes they would recall on each trial. Here, the cue period was replaced with a decision period, during which the participant decided which of the three memories they would subsequently recall. The same procedure as cued recalled was then followed, with the addition that after the recall period, participants were required to indicate via the button-box which of the three memories they had just recalled (for pair-wise statistical dependencies of this free choice behavior see Table S1). Ratings of vividness and accuracy were again taken for each trial. This condition included a total of thirty trials, and participants were instructed to sample from the three memories, while avoiding the recall of the same memory twice in a row. Both experimental conditions were scanned in a single functional run, starting with the cued recall condition, with a thirty second rest period before the free recall condition. The decoding analysis described below yielded significantly above chance (all p<0.001) decoding results in all three anatomical regions for both the cued and free recall conditions when analysed separately. In the cued recall condition, mean hippocampal accuracy was 52.5% (SD=0.087), mean entorhinal cortex accuracy was 44% (SD=0.086), and mean parahippocampal gyrus accuracy was 49.1% (SD=0.074). In the free recall condition, mean hippocampal accuracy was 49% (SD=0.055), mean entorhinal cortex accuracy was 46.1% (SD=0.048), and mean parahippocampal gyrus accuracy was 47.1% (SD=0.061). Furthermore, a

direct comparison of each region's accuracy values across the two conditions failed to find any significant differences in the hippocampus (p=0.189), entorhinal cortex (p=0.545), or parahippocampal gyrus (p=0.58), demonstrating that decoding does not depend on the specific retrieval mode. Therefore, for all subsequent analyses, the data were collapsed across both conditions in order to investigate patterns of information that hold across different retrieval modes. Table S2 summarises the behavioral performance of the participants during scanning.

Univariate Analysis

A standard mass univariate statistical analysis was performed using SPM8 (www.fil.ion.ucl.ac.uk/spm). Spatial preprocessing consisted of realignment and normalization to a standard EPI template in Montreal Neurological Institute (MNI) space, and smoothing using a Gaussian kernel with FWHM of 8mm. After preprocessing, statistical analysis was performed using the general linear model. Each of the three memories was modelled as a separate regressor, where the recall period of each trial was modelled as a boxcar function and convolved with the canonical hemodynamic response function. Participant-specific movement parameters were included as regressors of no interest. Participant-specific parameter estimates pertaining to each regressor (betas) were calculated for each voxel. These parameter estimates were entered into a second level random-effects analysis using a one-way ANOVA, with the three memory regressors as the three factors in the ANOVA. Given our *a priori* interest in the medial temporal lobes, a significance threshold of $p<0.001$, uncorrected for multiple comparisons, was employed for voxels within this region. A significance threshold of $p<0.05$ corrected for family-wise errors was employed for voxels elsewhere in the partial volume. No significant differences in activity were detected. These null univariate results were expected because the conventional univariate approach works by measuring gross voxel activity differences between conditions. With all conditions involving identical processes (episodic retrieval), it is not surprising that this method did not reveal any significant differences, hence the advantage of using a multivariate approach. We also conducted an additional univariate analysis comparing overall activity during memory recall (collapsed across all three memories) with baseline activity. This analysis specifically looked at overall signal change during episodic recall regardless of the specific memory. Even at a liberal threshold of p=0.001 uncorrected for multiple comparisons, there was no activity, for example, in the anterior hippocampus. This shows that the decoding results cannot merely be due to generally increased signal in this region during recall.

Image Preprocessing for Multivariate Analysis

T1-weighted structural images were put through the FreeSurfer [1-2] processing pipeline in order to generate a set of anatomical regions of interest (ROIs). FreeSurfer automatically assigns an anatomical label to each voxel based on a probabilistic atlas, and the technique has been shown to be comparable in accuracy to manual labelling [1-2]. This generated a set of hippocampus, entorhinal cortex, and parahippocampal gyrus masks for each participant. The anterior and posterior boundaries of the entorhinal and parahippocampal masks were altered manually where necessary to ensure that they were in line with the anatomical guidelines set out by Insausti et al. [3].

The first six EPI volumes were discarded to allow for T1 equilibration effects [4]. The remaining EPI images were then realigned to correct for motion effects, and minimally smoothed with a 3mm FWHM Gaussian kernel. A linear detrend was run on the images to remove any noise due to scanner drift [5]. Next the data were convolved with the canonical hemodynamic response function (HRF) to increase the signal-to-noise ratio [4]. This HRF convolution effectively doubled the natural BOLD signal delay, giving a total delay of approximately 12s. To compensate for this delay, all onset times were shifted forward in time by three volumes, yielding the best approximation to the 12s delay given a TR of 3.5s and rounding to the nearest volume [6]. Functional volumes were extracted from the vivid recall period of each trial, leading to a total of between two and four functional volumes per trial, depending on the precise start-time and length of the recall period in each case.

Multivariate Classification

In order to assess the degree of episodic information contained within MTL structures we used a two-step procedure incorporating first feature selection and then final multivariate classification [7]. The purpose of feature selection is to reduce the set of features (in this case, voxels) in a data set to those most likely to carry relevant information. This is effectively the same as removing voxels most likely to carry noise, and is a way of increasing the signal-to-noise ratio. Feature selection can therefore greatly improve the

performance of multivariate pattern classification [7]. The particular feature selection strategy employed was a multivariate searchlight strategy, which assesses the local pattern of information surrounding each voxel in turn [8-9] (see feature selection section below for more details). The overall classification procedure involved splitting the imaging data into two segments: a “training” set used to train a linear support vector machine (SVM) [10] (with fixed regularization hyperparameter C = 1) in order to identify response patterns related to the memories being discriminated, and a “test” set used to independently test the classification performance. Prior to multivariate classification, feature selection was performed on the data from the training set. This step produced a subset of voxels within the hippocampus (or in entorhinal cortex or parahippocampal gyrus) that contained the greatest degree of episodic decoding information within the training data set. Using this voxel subset, the SVM classifier was trained to discriminate between the three memories using the “training” image data set, and tested on the independent “test” data set (see Figure S1). The classification was performed with a SVM by using the LIBSVM (http://www.csie.ntu.edu.tw/~cjlin/libsvm/) implementation. We used a standard k-fold cross-validation testing regime [10] wherein k equalled the number of experimental trials, with the data from each trial set aside in turn as the test data, and the remaining data used as the training set (on each fold, the feature selection step was performed using only data from this training set). This therefore generated k sets of SVM training and test sets which produced an overall classification accuracy from the proportion of correct classification “guesses” across all k folds of the cross-validation.

Note that standard SVMs are binary classifiers that operate on two-class discrimination problems, whereas our data involved a three-class problem. The SVM can, however, be arbitrarily extended to work in cases where there are more than two classes. Typically this is done by reducing the single multiclass problem into multiple binary classification problems that can be solved separately and then recombined to provide the final class prediction [11]. We used the well-established Error Correcting Output Codes approach [12] to assign a unique binary string to each of the three classes. The length of the binary string corresponds to the number of binary classifiers performed. As there are 3 possible pair-wise comparisons that can be made between the three memories, the unique binary string “code words” were 3 bits in length. The 3 possible binary classifications were performed in each case, and their outputs combined into a 3-bit output code, with each bit representing the output from a single binary classifier. These output codes were then compared against all 3 of the preassigned class code words to determine the final predicted class. This was achieved by computing the Hamming distance [13] (i.e. the number of bits which differ between two binary strings) between the output code and the class code words to find the closest fit. The memory represented by this code word was then chosen as the output of the classification.

Feature Selection

Feature selection was implemented using a multivariate searchlight strategy [8], which examines the information in the local spatial patterns surrounding each voxel within the search space. Thus, for each voxel within the chosen anatomical region of interest, we investigated whether its local environment contained information that would allow accurate decoding of the three memories. For a given voxel, we first defined a small sphere with a radius of three voxels centred on the given voxel. This radius was chosen because a previous demonstration of hippocampal decoding using the searchlight method used radius three [9]. Note that the “spheres” were restricted so that only voxels falling within the given region of interest were included. Therefore the shape of the “sphere”, and the number of voxels within it varied depending on the proximity to the region of interest’s borders.

A linear SVM was then used in order to assess how much episodic information was encoded in these local pattern vectors (See Figure S2). This was achieved by splitting the feature selection data set into a training set and a test set (again it is important to note that all of the data used in this feature selection step is derived from the *training* set of the overall classification procedure, and therefore is fully independent of the final classification). The training set was then used to train a SVM classifier using the LIBSVM (http://www.csie.ntu.edu.tw/~cjlin/libsvm/) implementation and a fixed regularization hyperparameter of C = 1. We used a standard k-fold cross-validation testing regime [10] wherein k equalled the number of experimental trials minus one (as one trial is already removed for use as the overall testing set – see above), with the data from each trial set aside in turn as the test data, and the remaining data used as the training set. This therefore generated k sets of SVM training and test sets which produced an overall classification accuracy from the proportion of correct classification “guesses”

across all k folds of the cross-validation. This procedure was repeated for each searchlight sphere, thus generating a percentage accuracy value for every single voxel within the search space.

The searchlight analysis described above therefore produces an “accuracy map” of the given ROI, with an accuracy value at each voxel representing the amount of decoding information contained within the searchlight sphere surrounding that voxel. This allows us to perform feature selection by selecting searchlight spheres with high accuracy values. In this case, the searchlight with the maximal accuracy value was chosen as the output of feature selection. In cases where more than one searchlight carried the maximal accuracy value, all voxels from all the maximal searchlight spheres were included as the feature selection output.

Information Maps

The multivariate pattern analysis technique uses a feature selection procedure in order to select subsets of voxels more likely to carry information. This means that for each fold of the k-fold cross-validation, a different subset of voxels is selected. In order to visualise the voxels selected during feature selection, an “information map” was created by simply finding all voxel sets which produced above-chance accuracy on that particular cross-validation fold. These voxel sets were added together to form a single binary mask. Hippocampal information maps for all ten participants are displayed in Figure 3.

Overlap Analysis

To investigate the consistency of location of decoding across participants, the individual hippocampal information maps were normalized using the FreeSurfer high-dimensional warps previously generated during creation of the anatomical ROIs. The ten information maps could then simply be added together to form a frequency heat map. These heat maps are displayed in Figure 4. Assuming that the voxel location of individual information maps follows a binomial distribution, the likelihood of finding the same voxel by chance N times out of 10 was assessed for each voxel frequency value N, where in each hemisphere the maximum frequency was 6. For the left hippocampus, frequency levels of 5 and 6 survived an uncorrected threshold of $p = 0.001$, with one-tailed p values of 0.00056 and 0.00005 respectively. In the right hippocampus, a frequency level of 5 and 6 survived this threshold at $p = 0.0014$ and $p = 0.0002$ respectively.

Temporal Dependencies: Control Analysis

For any classification study, it is important to ensure that the data used for testing is independent of that used for training. In the current study the temporal gap between each recall period was at least 10s, which should ensure that the testing and training data are relatively independent. However, to test this assumption, we conducted a control analysis where we increased the temporal gap between the testing and training data. If residual temporal dependencies were affecting the results, then this increased temporal gap should significantly impair classification performance. This analysis was identical to the main analysis, but on each fold of the k-fold cross-validation, the trials that were temporally adjacent to the testing trial (trials k-1 and k+1) were excluded from both the feature selection data and the training data. This effectively increased the temporal gap between training and testing data to at least 26s. We found significant episodic decoding in all three MTL regions, with mean hippocampus accuracy of 44% ($p = 0.000001$; chance level = 33%), mean entorhinal cortex accuracy of 38.5% ($p = 0.009$), and mean parahippocampal gyrus accuracy of 41% ($p = 0.0004$). A direct comparison of these new results with the original results did not find significant differences for any of the three MTL regions. These results demonstrate that the addition of a substantial temporal gap between testing and training data does not make any significant difference to the decoding performance, and we can therefore be confident that our training and testing data are independent.

Comparison of Cued and Free Recall Conditions

To ensure that the decoding results were based on information that was consistent across the two modes of retrieval, we performed a further control analysis. A searchlight classifier was applied to the hippocampi using only the free recall data. The maximal searchlight was found, and this set of voxels was then used to train on the free recall data and test on the cued recall data. Given the large reduction in training data that results from this procedure, we would expect a considerable loss in classifier sensitivity via this approach. Nevertheless, collapsing across both hippocampi there was a trend towards significant decoding (mean accuracy 35%, $p = 0.12$; chance = 33%) with a significant result in the right hippocampus

(mean accuracy 38%, $p = 0.028$). These results demonstrate that the classifier is making use of common information across the different conditions and does not rely on information specific to the mode of retrieval.

Debriefing Questionnaire
After the scanning session, participants answered a debriefing questionnaire, which was designed to assess aspects of their memory recall. They were asked to provide ratings (on a scale of 1 – 5, low - high) for each of the three memories based on the average response across all trials during scanning for the following:

How hard did you find it to vividly recall this event?

How emotional did this event make you feel?

How much did this event make you think about a real memory from your own life?

How much did this event make you think about yourself?

How much did you find yourself thinking about some sort of background story behind the event?

How much did you find yourself trying to take the perspective of the person in these events?

There were no significant differences between the three memories for any of these ratings (see Table S3). Additionally, participants were asked whether they recognised the person or location featured in each event, and to give a rating (1-5, low-high) of their general attention during scanning. No participants recognised the people or places. The mean rating of attention was 4.1 (SD 0.57).

Supplemental References

1. Fischl, B., Salat, D.H., Busa, E., Albert, M., Dieterich, M., Haselgrove, C., van der Kouwe, A., Killiany, R., Kennedy, D., Klaveness, S., et al. (2002). Whole brain segmentation: automated labelling of neuroanatomical structures in the human brain. Neuron *33*, 341-355.
2. Fischl, B., van der Kouwe, A., Destrieux, C., Halgren, E., Segonne, F., Salat, D.H., Busa, E., Seidman, L.J., Goldstein, J., Kennedy, D., et al. (2004). Automatically parcellating the human cerebral cortex. Cereb. Cortex. *14*, 11-22.
3. Insausti, R., Juottonen, K., Soininen, H., Insausti, A.M., Partanen, K., Vainio, P., Laakso, M.P., Pitkanen, A. (1998). MR volumetric analysis of the human entorhinal, perirhinal, and temporopolar cortices. AJNR Am. J. Neuroradiol. *19*, 659-71.
4. Frackowiak, R.S.J., Friston, K.J., Frith, C.D., Dolan, R.J., Price, C.J., Zeki, S., Ashburner, J.T., Penny. W.D. (2004). Human Brain Function (New York: Elsevier Academic Press).
5. LaConte, S., Strother, S., Cherkassky, V., Anderson, J., Hu, X. (2005). Support vector machines for temporal classification of block design fMRI data. Neuroimage *26*, 317-329.
6. Haynes, J. D., Rees, G. (2006). Decoding mental states from brain activity in humans. Nat. Rev. Neurosci. *7*, 523-534.
7. Guyon, I., Elisseeff, A. (2003). An introduction to variable and feature selection. Journal of Machine Learning Research *3*, 1157-1182.
8. Kriegeskorte, N., Goebel, R., Bandettini, P. (2006). Information-based functional brain mapping. Proc. Natl. Acad. Sci. U.S.A. *103*, 3863-3868.
9. Hassabis, D., Chu, C., Rees, G., Weiskopf, N., Molyneux, P.D., Maguire, E.A. (2009). Decoding neuronal ensembles in the human hippocampus. Curr. Biol. *19*, 546-54.
10. Duda, O.R., Hart, P.E., Stork, D.G. (2001). Pattern Classification (New York: Wiley).
11. Allwein, E., Schapire, R., and Singer, Y. (2000). Reducing multiclass to binary: a unifying approach for margin classifiers. Journal of Machine Learning Research *1*, 113-141.
12. Dietterich, T.D., and Bakiri, G. (1994). Solving multiclass learning problems via error-correcting output codes. Journal of Artificial Intelligence Research *2,* 263–286.
13. Hamming, R.W. (1950). Error-detecting and error-correcting. Bell System Technical Journal *29*, 147-160.
